# Special Issue: Molecular Research on Depression

**DOI:** 10.3390/ijms26020643

**Published:** 2025-01-14

**Authors:** Magdalena Sowa-Kućma, Katarzyna Stachowicz

**Affiliations:** 1 Department of Human Physiology, Faculty of Medical Sciences, Medical College of Rzeszów University, Kopisto 2a, 35-315 Rzeszów, Poland; 2 Centre for Innovative Research in Medical and Natural Sciences, Medical College of Rzeszów University, Warzywna 1a, 35-310 Rzeszów, Poland; 3 Department of Neurobiology, Maj Institute of Pharmacology, Polish Academy of Sciences, Smetna 12, 31-343 Krakow, Poland; stachow@if-pan.krakow.pl

Depressive disorders (DDs) are responsible for a significant burden of disease in the human population. This problem affects not only individuals, but also family members and society. Therefore, it is a severe social and economic problem in the 21st century [1,2]. For several decades, many theories (noradrenergic, serotonergic, dopaminergic, or glutamate transmission disturbances) of depression have been formulated on which modern pharmacotherapy is based [3,4]. Unfortunately, 30% (or even more) of depressive patients do not respond to traditional treatment and first-line treatment options [5]. The key to developing new, more effective therapeutic strategies is to understand the molecular mechanisms underlying DDs [6]. The authors of this Special Issue offer new solutions and insights into the subject and present the most recent research in search for mechanisms and biomarkers of depression. This Special Issue of the International Journal of Molecular Sciences, entitled “Molecular Research on Depression”, comprises a total of seven contributions: six original articles and one review providing the latest research findings on DDs.

Microglia activation is one of the most commonly studied pathways in this area. The nod-like receptor family represents a novel candidate microglia expressed in depression [7]. Nucleotide-binding oligomerization domain-like receptor family caspase recruitment domain-containing five receptor (NLRC5) deficiency has been shown to inhibit microglia activation in the mouse hippocampus and improve depressive behavior in two models of depression, i.e., lipopolysaccharide-mediated and chronic unpredictable mild stress [1]. Another important marker of inflammation in depression is macrophage migration inhibitory factor (MIF) [7]. A recent study in patients with a major depressive episode revealed the absence of a homozygous minor allele as protective in women with major depressive disorder (MDD). However, similar effects were not observed in men [7]. The relationship between depression and inflammation was also studied by Saito-Takatsuji et al. [7]. Chronic stress induced the development of depression-like behaviors in animals, which was accompanied by an increase in the expression of Transthyretin (Ttr) in the hippocampus. Conversely, the overexpression of Ttr in the hippocampus led to an increase in the expression of Icam1 and Vcam1, genes associated with inflammation [7]. Continuing the theme, Kirchner et al. [7] described the effects of various genetic factors (e.g., APOE ε4, BDNF Val, and 5-HTTLPR L alleles) on hippocampal subfield volumes in the general population related to, among other factors, depression or memory impairment. The influence of genetics on the pharmacotherapy of depression was discussed in a paper by Zieba et al. [7]. In turn, Brazdis et al. [7] draw attention to the co-occurrence of Parkinson’s disease and MDD, and suggest that an analysis of the expression of genes (α-synuclein, lysosomal enzyme β-glucosacerebrosidase, and UDP-glucose ceramide glucosyltransferase) closely related to PD may be helpful in searching for biomarkers of MDD and understanding the pathomechanisms associated with neuropsychiatric diseases. A slightly different but very interesting perspective on the search for predictive markers of depression was presented by Kasuya et al. [7], who conducted research on participants of the Japanese Antarctic Research Expedition. Some participants had major depressive episodes consistent with altered metabolic profiles (including ornithine and beta-alanine levels). The most significant differences were observed after three months, when depressive episodes were not yet very severe. Therefore, it is suggested that changes in selected metabolites in urine may help to develop biomarkers to detect people with depressive episodes at an early stage of disease development [7].

In conclusion, this Special Issue contains the latest and most valuable information on the molecular basis of depressive disorders.

## References

[1] World Health Organization (WHO) Depression. https://www.who.int/news-room/fact-sheets/detail/depression.

[2] Tompson A., Alkasaby M., Choudhury T., Dun-Campbel K., Hartwell G., Körner K., Maani N., van Schalkwyk M.C.I., Petticrew M. (2024). Addressing the commercial determinants of mental health: An umbrella review of population-level interventions. Health Promot. Int..

[3] Samojedny S., Czechowska E., Pańczyszyn-Trzewik P., Sowa-Kućma M. (2022). Postsynaptic Proteins at Excitatory Synapses in the Brain-Relationship with Depressive Disorders. Int. J. Mol. Sci..

[4] More S., Kaleem M., Kharwade R., Almutairy A.F., Shahzad N., Ali Mujtaba M., Taha M., Pise A., Zafar A., Mahmood D. (2025). Depression unveiled: Insights into etiology and animal models for behavioral assessment, exploring the multifactorial nature and treatment of depression. Brain Res..

[5] Medeiros G.C., Demo I., Goes F.S., Zarate C.A., Gould T.D. (2024). Personalized use of ketamine and esketamine for treatment-resistant depression. Transl. Psychiatry.

[6] Stachowicz K., Sowa-Kućma M. (2022). The treatment of depression-searching for new ideas. Front Pharmacol..

[7] https://www.mdpi.com/journal/ijms/special_issues/Depression_Molecular.

